# Inhibition of c-Myc Overcomes Cytotoxic Drug Resistance in Acute Myeloid Leukemia Cells by Promoting Differentiation

**DOI:** 10.1371/journal.pone.0105381

**Published:** 2014-08-15

**Authors:** Xiao-Na Pan, Jia-Jie Chen, Le-Xun Wang, Ruo-Zhi Xiao, Ling-Ling Liu, Zhi-Gang Fang, Quentin Liu, Zi-Jie Long, Dong-Jun Lin

**Affiliations:** 1 Department of Hematology, Third Affiliated Hospital, Sun Yat-sen University, Guangzhou, China; 2 Sun Yat-sen Institute of Hematology, Sun Yat-sen University, Guangzhou, China; Peking University Health Science Center, China

## Abstract

Nowadays, drug resistance still represents a major obstacle to successful acute myeloid leukemia (AML) treatment and the underlying mechanism is not fully elucidated. Here, we found that high expression of c-Myc was one of the cytogenetic characteristics in the drug-resistant leukemic cells. c-Myc over-expression in leukemic cells induced resistance to chemotherapeutic drugs, enhanced colony formation capacity and inhibited cell differentiation induced by all-trans retinoic acid (ATRA). Meanwhile, inhibition of c-Myc by shRNA or specific c-Myc inhibitor 10058-F4 rescued the sensitivity to cytotoxic drugs, restrained the colony formation ability and promoted differentiation. RT-PCR and western blotting analysis showed that down-regulation of C/EBPβ contributed to the poor differentiation state of leukemic cells induced by c-Myc over-expression. Importantly, over-expression of C/EBPβ could reverse c-Myc induced drug resistance. In primary AML cells, the *c-Myc* expression was negatively correlated with *C/EBPβ*. 10058-F4, displayed anti-proliferative activity and increased cellular differentiation with up-regulation of C/EBPβ in primary AML cells. Thus, our study indicated that c-Myc could be a novel target to overcome drug resistance, providing a new approach in AML therapy.

## Introduction

Acute myeloid leukemia (AML) is a kind of aberrant clonal hematopoietic malignancy originating from genetic mutations, or epigenetic aberrations in normal hematopoietic progenitors. Although chemotherapy results in clinical remission for AML, drug resistance still represents a huge challenge to achieve effective treatment, especially in relapsed or refractory AML patients [1], [2]. Cytostatic drug resistance occurs through different mechanisms, including induction of drug detoxification [3], the over-expression of oncogene or inactivation of tumor suppressor gene [4], [5], metabolic disturbance [6], [7], as well as existence of leukemia stem cells [8]. Still, the exact mechanisms of drug resistance in leukemia have not been fully investigated. Elucidation of the intrinsic or acquired factors that mediate drug resistance remains of critical importance for the development of novel therapeutic strategies for relapsed or refractory AML patients.

The proto-oncogene *c-Myc* plays a pivotal role in cellular metabolism [9], apoptosis [10], differentiation [11], cell cycle progress [12], and tumorigenesis [13]. *c-Myc* encodes a basic helix-loop-helix leucine zipper transcription factor, which transcripts an array of downstream target genes [14]. c-Myc, as an attractive target for cancer therapy, is aberrantly expressed in a wide variety of human solid tumors [15] as well as leukemia [16]. Study reported that c-Myc over-expression was closely correlated to chemotherapy resistance in salivery carcinoma [17]. Inhibition of c-Myc overcame drug resistance in some cancers, such as Lewis lung carcinoma [18] and melanoma [19]. *c-Myc* antisense oligodeoxynucleotides increased cisplatin sensitivity in metastatic melanoma cell lines inherently resistant to cisplatin [20]. 10058-F4, a targeted inhibitor of c-Myc, was reported to be effective in anti-tumor treatment, such as hepatocellular carcinoma [21] and leukemia [22]. However, the precise role of c-Myc in drug resistance of leukemic cells has not yet been elucidated.

In this study, we identified the effects of c-Myc on drug resistance in leukemic cell lines and AML primary cells. We found that the up-regulated expression of c-Myc in leukemic cells promoted colony formation ability and maintained poor differentiation mediated by suppression of C/EBPβ, leading to drug resistance. Consistently, down-regulation of c-Myc abrogated colony formation capacity of leukemic cells and promoted cellular differentiation. Our study provided a new approach to overcome drug resistance by c-Myc inhibition in AML therapy.

## Materials and Methods

### Primary AML cell isolation

AML patient samples were obtained with the written informed consent in accordance with the Declaration of Helsinki and the approval by the Medical Ethical Committee of the Third Affiliated Hospital of Sun Yat-sen University. Bone marrow mononuclear cells (BMMCs) were enriched by Ficoll-Hypaque density gradient centrifugation. Refractory and relapsed (R) AML samples was included on the basis of the Chinese edition of NCCN Guidelines (Version 2011).

### Cell culture

Primary leukemia BMMCs were resuspended in RPMI 1640 medium (Gibco, Grand Island, NY, USA) containing appropriate antibiotics and 10% fetal bovine serum (FBS, HyClone, Logan, UT, USA). K562 and U937 cell lines were purchased from American Type Culture Collection (ATCC, Manassas.VA, USA). NB4 and drug-resistant NB4-R2 cells were provided by Shanghai Institute of Hematology, Ruijin Hospital. Imatinib (Gleevec) resistant K562 cell line (K562/G) was the gift of Prof. Wen-Lin Huang, Cancer Center of Sun Yat-sen University.

### Cell cytotoxicity Assay

Cell cytotoxicity was evaluated by MTT assay according to the manufacture's instruction. Briefly, leukemic cells or primary BMMCs were seeded in the 96-well U-bottomed plate. Subsequently, cells were treated with different drugs at different concentrations for indicated time. After MTT solution was added to each well, cells were incubated at 37°C for another 4 h and the absorbance was finally determined at 490 nm using the microplate reader (BioTek, Vermont, USA).

### Colony formation assay

Cells with different drugs treatment were plated in 10% FBS medium with methylcellulose (R&D Systems, Minneapolis, MN, USA) for 6 days. The colony forming units (CFU) were counted using microscopy (IX81, Olympus, Japan), and the colony number was the sum of randomized forty visions.

### Retroviral and lentiviral transduction

The MSCV 2.2 vector was kindly provided by Prof. Hua Huang (University of Colorado, Denver, USA). The *c-Myc* and *C/EBPβ* gene were cloned and inserted into the MSCV 2.2 vector. Retrovirus was produced from packaging cell line GP2-293 with the aid of pseudo-envelope vector. Lentiviral vector expressing short hairpins against human *c-Myc* (ShMYC) was constructed within the lentivirus plasmid vector pLL3.7 using the target sequence 5′-CCATAATGTAAACTGCCTCAA-3′. Lentiviral particles were produced by cotransfection of 293T cells with packaging vectors (pMD2.G and pPAX2). The virus supernatants were collected 48 h after transfection.

### Cell morphology analysis

Cells were seeded into 6-well plates and incubated with specific drug for indicated time and collected for analysis. Cytospin slides were prepared, and the cells were counterstained with Wright-Giemsa. The morphology of cells was observed by microscopy (7×41, Olympus, Japan).

### Western blotting analysis

Total cellular proteins were isolated with lysis buffer. Equal amounts of protein were subjected to SDS-PAGE and transferred to nitrocellulose membranes. The membranes were blocked and then incubated with c-Myc (Santa Cruz Biotechnology, Santa Cruz, CA, USA), C/EBPβ (Abcam, Cambridge, UK) and GAPDH antibody (Cell Signaling Technology Corp, Beverly, MA, USA). The protein bands were visualized using an enhanced chemiluminescence reagent (Sigma, St. Louis, MO, USA), according to the manufacturer's instruction.

### Flow cytometry analysis

Analysis of CD11b expression was performed using the PE anti-human CD11b antibody (BD Biosciences, San Jose, CA, USA) for predicting the cellular differentiation.

### Nitrotetrazolium blue chloride (NBT) reduction assay

The same amount of cells untreated or treated with ATRA for 72 h were incubated at 37°C for 1 h in RPMI 1640 medium containing 10% FBS and 0.1% NBT (Sigma-Aldrich, MO, USA). The cells were then centrifuged and dissolved in DMSO. The optical density was read at 570 nm.

### RNA extraction and PCR analysis

RNA was isolated using Trizol reagent (Invitrogen, Grand Island, NY, USA), and reverse transcribed using M-MuLV reverse transcriptase (Fermentas, St Leon-Roth, Germany). Real-time PCR was performed using SYBR Green qPCR mix (Toyabo, Osaka, Japan) on Applied Biosystems 7500 Fast Real-Time PCR thermocycler (Applied Biosystems, Foster City, CA, USA) and quantified using ABI 7500 software.

### Statistical analysis

Data were presented as mean ± SD. Statistical analysis was performed using SPSS v. 16.0 (SPSS, Inc.) and GraphPad Prism 6 (GraphPad Software, Inc.). A value of p<0.05 was considered statistically.

## Results

### c-Myc is high expressed in drug resistant leukemic cells

**Figure 1:**
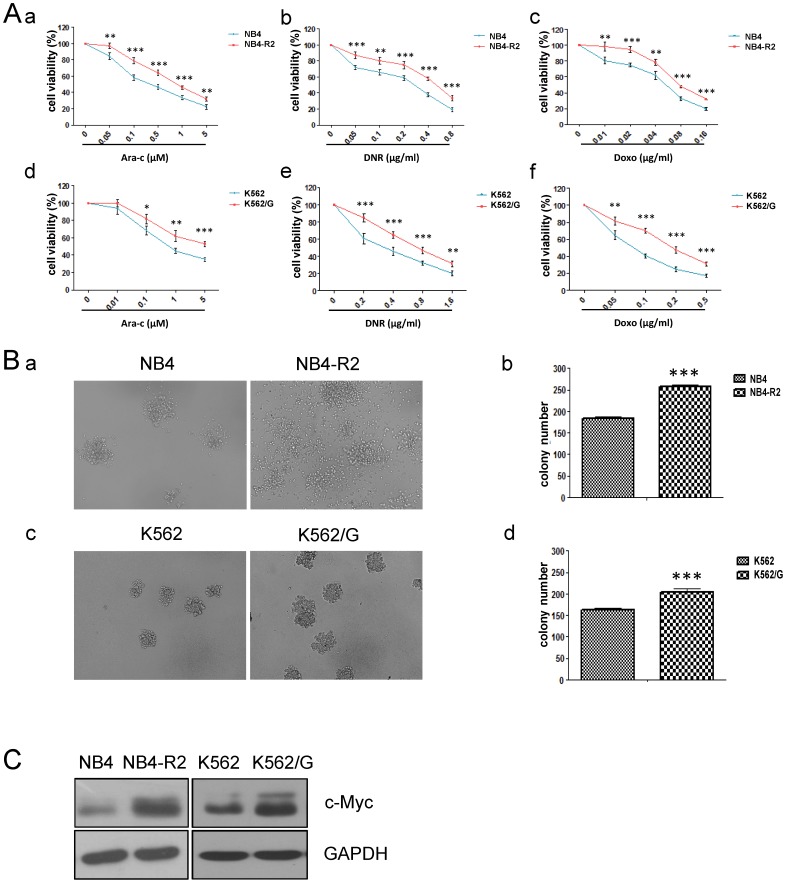
c-Myc is high expressed in drug resistant leukemic cells. (A) Drug-resistant cells (NB4-R2 and K562/G) and drug-sensitive cells (NB4 and K562) were treated with various concentrations of Ara-C, DNR or Doxo for 48 h and subjected to MTT assay. (B) The colony formation images of drug-resistant and -sensitive cells were obtained using a light microscope (magnification, ×200) under 6 days' culture (a,c), and the statistical data were followed (b,d). (C) Western blotting was performed to analyze c-Myc expression. Cell lysates were incubated with antibodies against c-Myc and GAPDH as indicated. Data summarized three independent experiments. *p<0.05, **p<0.01, ***p<0.001, Student's t test.

To find the cytogenetic characteristics in drug-resistant leukemic cells, two drug-resistant leukemic cell lines were applied for detecting the properties. NB4-R2 and K652/G were respectively resistant to all-trans-retinoic acid (ATRA) and imatinib, compared with their parental cell lines (NB4 and K562), as shown in Figure S1. The anti-proliferative effect of cytarabine (Ara-C), daunomycin (DNR) or doxorubicin (Doxo), clinical cytotoxic drugs for treating AML, was detected by MTT assay (Figure 1A). The IC50 values of Ara-C, DNR or Doxo at 48 h were 0.3900 µM, 0.2097 µg/ml or 0.0469 µg/ml respectively for NB4, 1.4770 µM, 0.4540 µg/ml or 0.0838 µg/ml for NB4-R2, 0.8010 µM, 0.3325 µg/ml or 0.0827 µg/ml for K562, and 6.0368 µM, 0.7375 µg/ml or 0.2063 µg/ml for K562/G. The result showed that NB4-R2 and K652/G were both resistant to Ara-C, DNR or Doxo, indicating that the two resistant leukemic cells had a broad-spectrum of resistance to clinical cytotoxic drugs. The colony number of NB4-R2 cells was more than that of NB4 cells under 6 days' culture (Figure 1B-a). Similar phenomenon was observed in K562/G cells compared to K562 cells (Figure 1B-b). The data indicated drug-resistant cell lines also displayed increased colony formation capacity. Chemotherapy resistance was closely correlated to *c-Myc* amplification, we thus testify whether c-Myc over-expression was involved in the drug-resistant leukemic cells. Western blotting assay revealed that the expression level of c-Myc was higher in drug-resistant cells (NB4-R2 and K562/G) than in the control cells (Figure 1C), suggesting that high c-Myc expression was correlated with enhanced drug-resistance and colony formation capacity in leukemic cells.

### c-Myc over-expression contributes to drug resistance and high colony formation capacity in leukemic cells

**Figure 2:**
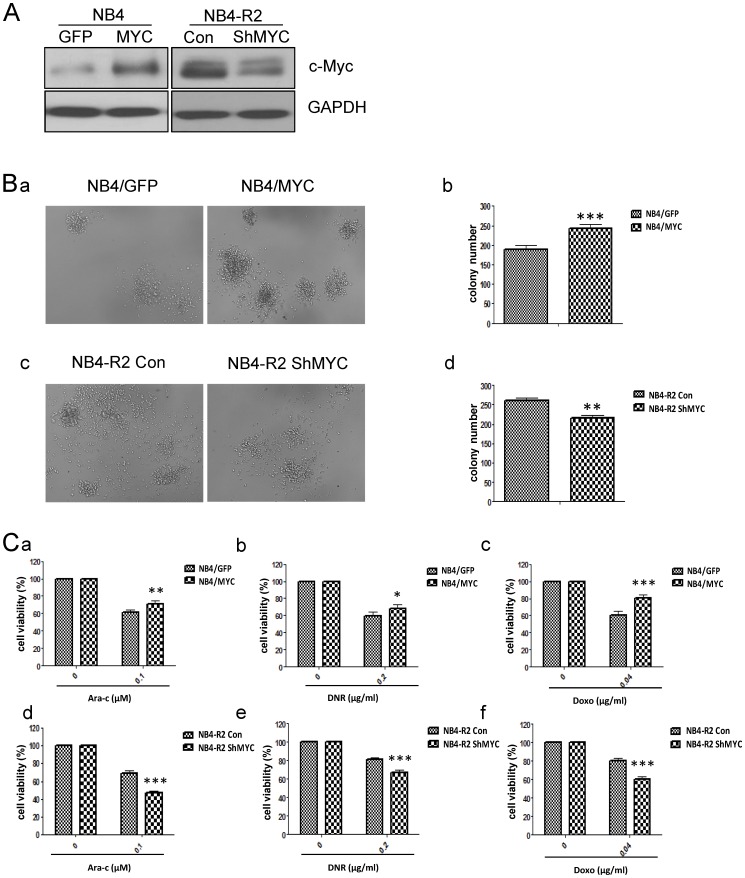
c-Myc over-expression contributes to drug resistance and high colony formation capacity in leukemic cells. (A) c-Myc was over-expressed in NB4 cells (NB4/MYC), and was knocked down in NB4-R2 cells (NB4-R2 ShMYC). Cell lysates were subjected to western blotting analysis. (B) The colonies were photographed under the light microscope (magnification, ×200; a,c), and the statistical results were shown (b,d). (C) The stable leukemic cell lines with up- or down-expression of c-Myc were treated with Ara-C (0.1 µM), DNR (0.2 µg/ml) or Doxo (0.04 µg/ml) for 48 h. Drug sensitivity was testified by MTT assay. Data summarized three independent experiments. *p<0.05, **p<0.01, ***p<0.001, Student's t test.

In order to identify the effect of c-Myc on drug resistance and colony formation in leukemia, we constructed the stable leukemic cell line with c-Myc over-expression or knock-down for colony formation assay and drug sensitivity test. The stable leukemic cell line with c-Myc over-expression (NB4/MYC) was derived from the drug-sensitive NB4 cells infected with MSCV retrovirus with *c-Myc* gene while NB4 cells with MSCV-GFP vector was used as the control (NB4/GFP). Lentiviral vector expressing short hairpins against human *c-Myc* (ShMYC) was used to reduce c-Myc expression in drug-resistant cell line NB4-R2 (NB4-R2 ShMYC), while lentiviral virus with the non-target ShRNA acted as the control (NB4-R2 Con). Western blotting showed that c-Myc expression was increased in NB4/MYC cells, while decreased in NB4-R2 ShMYC (Figure 2A). Next, the colony formation assay showed that c-Myc over-expression enhanced the colony formation ability in NB4 cells, and that reduced c-Myc expression restrained the colony formation in drug-resistant cells NB4-R2, as shown in Figure 2B. Drug sensitivity test based on MTT assay was performed to testify the cytotoxic effect with Ara-C, DNR or Doxo treatment when up- or down-regulation of c-Myc in leukemic cells. Cells were treated with Ara-C (0.1 µM), DNR (0.2 µg/ml) or Doxo (0.04 µg/ml) for 48 h. The rate of cell viability of NB4/GFP vs NB4/MYC in treatment with the indicated concentration of Ara-C, DNR or Doxo was 61.38%±3.26% vs 70.86%±3.75%, 59.82%±4.51% vs 68.37%±4.13%, or 60.85%±4.85% vs 80.64%±3.83% respectively, while the rate of NB4-R2 Con vs NB4-R2 ShMYC was 69.57%±2.12% vs 47.41%±1.74%, 80.89%±2.38% vs 67.19%±2.14%, or 80.70%±2.32% vs 60.21%±2.37% respectively, as shown in Figure 2C. Thus, increased c-Myc expression in NB4 cells promoted drug resistance to Ara-C, DNR or Doxo. In contrast, decreased c-Myc expression in drug-resistant cells NB4-R2 rescued the sensitivity to the cytotoxic drugs. Similar phemonemon was also observed in U937 cells (Figure S2A–2C).

### c-Myc inhibits cell differentiation induced by ATRA

**Figure 3:**
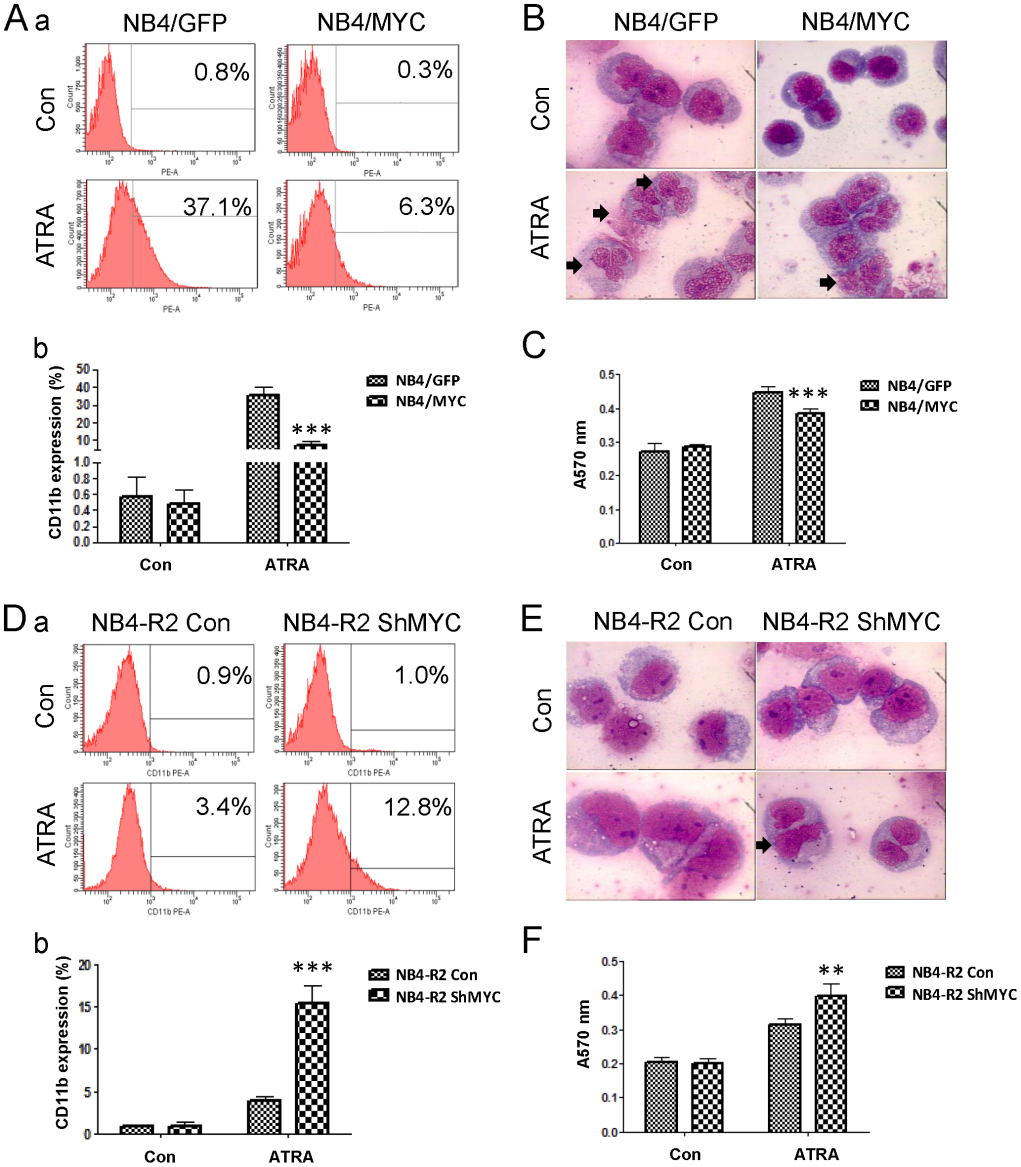
c-Myc inhibits cell differentiation induced by ATRA. NB4/GFP and NB4/MYC, as well as NB4-R2 Con and NB4-R2 ShMYC, were treated with 1 µM ATRA for 72 h. (A, D) Cells were then subjected to flow cytometry to determine the expression of CD11b (a), and the percentages of CD11b positive cells were under census (b). (B, E) Wright-Giemsa staining images of cells were captured by oil immersion lens (magnification, ×1 000). Segmented cells after 72 h of ATRA incubation were annotated by black arrows. (C, F) NBT reduction assay was performed to clarify the differentiation state. **p<0.01, ***p<0.001, Student's t test.

c-Myc acted as a key transcriptional regulator to maintain stemness in cancer stem like cells [23], [24]. c-Myc was also proposed to play a role in the transition from proliferation to differentiation in oligodendrocyte progenitor cells [25]. To explain the mechanism that high c-Myc expression induced drug resistance and promoted colony formation capacity in leukemic cells, we used the cell differentiation model of NB4 cells induced by ATRA. The result showed that the percentage of CD11b-positive cells induced by ATRA was significantly decreased from 35.8%±4.0% to 7.4%±2.0% after c-Myc over-expression in NB4 cells (Figure 3A). As well, NB4/GFP cells treated with ATRA represented nucleolus disappearance and obvious nuclear segmentation by Wright-Giemsa staining. However, this phenotype was reversed in NB4/MYC cells (Figure 3B). The retard of maturation resulting from c-Myc over-expression was also detected by NBT reduction assay (Figure 3C). Consistantly, c-Myc over-expression also inhibited U937 cells differentiation induced by ATRA (Figure S2D–2F). Contrarily, NB4-R2 ShMYC cells with reduced expression of c-Myc showed more CD11b-positive cells after ATRA treatment than the control cells (Figure 3D). The result was consistent with morphologic observation and NBT reduction assay (Figure 3E, 3F). Thus, the drug resistant cells were inclined to maintain poorly differentiated state resulting from elevated expression of c-Myc.

### Over-expression of C/EBPβ reverses c-Myc induced drug resistance

**Figure 4:**
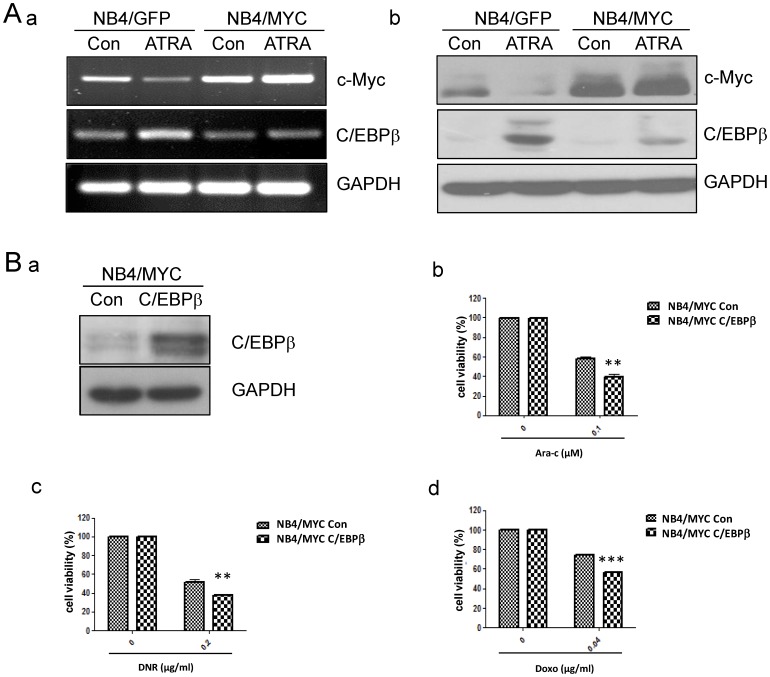
Over-expression of C/EBPβ reverses c-Myc induced drug resistance. (A) NB4/GFP and NB4/MYC cells were treated with 1 µM ATRA for 72 h. Expression of c-Myc, C/EBPβ was tested by RT-PCR and Western blot assay. GAPDH was used as an internal control. (B) C/EBPβ was over-expressed in NB4/MYC cells (NB4/MYC C/EBPβ). Western blotting analysis was performed to detect expression of C/EBPβ (a). NB4/MYC C/EBPβ cells were treated with Ara-C (0.1 µM), DNR (0.2 µg/ml) or Doxo (0.04 µg/ml) for 48 h. Drug sensitivity was testified by MTT assay (b–d). Data summarized three independent experiments. **p<0.01, ***p<0.001, Student's t test.

C/EBPβ was essential in the process of granulocytic differentiation. The DNA binding capacity and protein expression level of C/EBPβ were increased evidently in ATRA-induced differentiation of APL cells [26]. Here, we assessed whether high colony formation ability and dedifferentiation state of drug resistant leukemic cells induced by c-Myc was through manipulating C/EBPβ. RT-PCR and western blotting analysis were performed to further validate the expression of C/EBPβ. C/EBPβ was increased after ATRA treatment in NB4/GFP cells, but high c-myc expression inhibited the up-regulation of C/EBPβ in both mRNA and protein levels (35KD and 38KD isoforms) after ATRA treatment (Figure 4A). The data suggested that C/EBPβ was especially down-regulated by c-Myc in ATRA-induced differentiation. To testify the effect of C/EBPβ in drug resistance, C/EBPβ was over-expressed in NB4/MYC cells (NB4/MYC C/EBPβ) by transient retrovirus transduction (Figure 4B-a). MTT assay was carried out to detect the drug sensitivity of NB4/MYC C/EBPβ cells treated with Ara-C (0.1 µM), DNR (0.2 µg/ml) or Doxo (0.04 µg/ml). Result showed that C/EBPβ could rescue drug sensitiviy in NB4/MYC cells (Figure 4B-b, c, d), futher indiating that down-regulation of C/EBPβ was involved in drug resistance induced by c-Myc.

### c-Myc inhibitor 10058-F4 restrains drug resistance and colony formation ability induced by c-Myc over-expression

**Figure 5:**
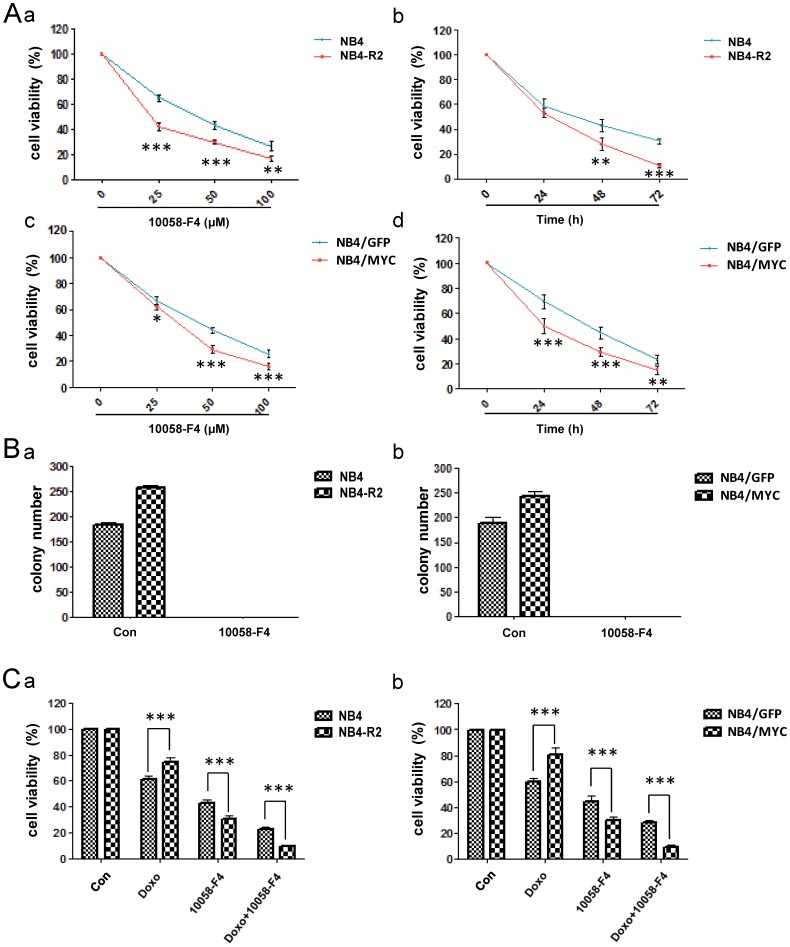
c-Myc inhibitor 10058-F4 restrains drug resistance and colony formation ability induced by c-myc over-expression. (A) High c-Myc expression cells (NB4-R2 and NB4/MYC) and the control cells (NB4 and NB4/GFP) were exposed to various concentrations of 10058-F4 for various times, and cell viability was determined by MTT assay. (B) The colony number of cells was noted by the column diagram. (C) The two group of cells were exposed to Doxo (0.04 µg/ml) and 10058 (50 µM) alone or combination. MTT assay was performed to testify the cell viability. Data summarized three independent experiments. *p<0.05, **p<0.01, ***p<0.001, Student's t test.

c-Myc was reported to dimerize with Max to play a part in gene transcription. 10058-F4, a small-molecule c-Myc inhibitor, specifically disrupts the c-Myc/Max heterodimerization, resulting in inhibiting the transcriptional function of c-Myc [27]. To clarify the effect of 10058-F4 on drug-resistant leukemic cells, MTT assay was performed to detect the anti-proliferative effect of 10058-F4. Result showed that the drug-resistant cell lines (NB4-R2 and NB4/MYC) were more sensitive to 10058-F4 than the drug-sensitive cell lines (NB4 and NB4/GFP) in both dose- and time-dependent manners (Figure 5A). We also found that the colony formation capacity of both drug-resistant and -sensitive cells were completely impeded by 10058-F4 (Figure 5B). In addition, 10058-F4 promoted the sensitivity of drug resistant cells responsing to Doxo treatment (Figure 5C). The data suggested that 10058-F4 could act as a new targeted therapeutic drug to improve the efficacy of chemotherapy in drug resistant leukemic cells.

### Inhibition of c-Myc suppresses proliferation and induces differentiation in primary AML cells

**Figure 6:**
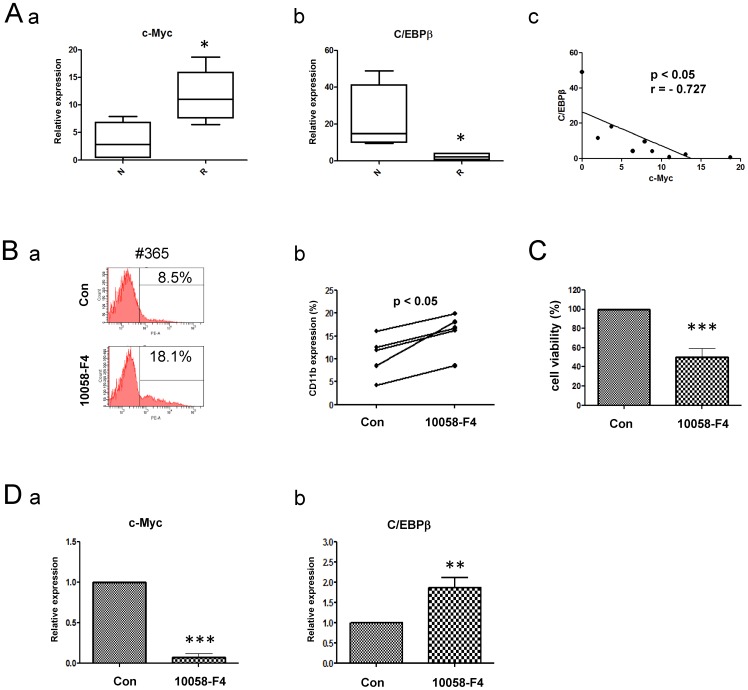
Inhibition of c-Myc suppresses proliferation and induces differentiation in primary AML cells. (A) BMMCs were collected from AML patients, which contained 5 refractory and relapsed (R) AML patients and 4 newly diagnosed (N) AML patients. Real time-PCR analysis was performed to clarify the mRNA expression of *c-Myc* (a) and *C/EBPβ* (b). The relative expression values from different patients were presented in a box plot graph. The horizontal line within each box represented the median value. The correlation between *c-Myc* and *C/EBPβ* expression was shown as (c). (B) BMMCs from five newly diagnosed AML patients were treated with 100 µM 10058-F4 for 48 h. The CD11b-positive cells was analyzed by flow cytometry (a), and the percentages of CD11b expression were under census (n = 5; b). (C) The anti-proliferation of 10058-F4 to BMMCs was detected by MTT assay. (D) The expression of *c-Myc* and *C/EBPβ* in BMMCs treated with 10058-F4 was detected by Real-time PCR. *p<0.05, **p<0.01, ***p<0.001, Student's t test.

To futher investigate the relation between c-Myc and C/EBPβ in primary AML cells, BMMCs from AML patients were collected, which contained 5 refractory and relapsed (R) AML patients and 4 newly diagnosed (N) AML patients. RT-PCR analysis showed that the expression of *c-Myc* was higher in refractory and relapsed patients than in newly diagnosed patients (Figure 6A-a). Meanwhile, *C/EBPβ* expression was significantly lower in refractory and relapsed patients (Figure 6A-b). Besides, the inverse correlation between *c-Myc* and *C/EBPβ* mRNA level was observed in primary AML cells (Figure 6A-c). Next, BMMCs from 5 AML patients were treated with 10058-F4, and flow cytometry was used to detect the differentiation state of primary AML cells. Our data showed that the percentage of CD11b-positive cells was increased after 10058-F4 treatment, illustrating that inhibition of c-Myc expression promoted the primary AML cells to differentiate to mature cells (Figure 6B). In the meantime, the cytotoxic effect of 10058-F4 was detected by MTT assay, and cell viability was reduced to 49.94%±8.99% after treatment (Figure 6C). In addition, *C/EBPβ* was up-regulated in primary AML cells treated with 10058-F4 (Figure 6D). Therefore, 10058-F4 could induce differentiation and retard the proliferation in primary AML cells.

## Discussion

Here, we addressed that up-regulation of c-Myc expression was one of cytogenetic characteristics in the drug-resistant leukemic cells with increased colony formation capacity (Figure 1). Over-expression of c-Myc in NB4 cells reduced the drug sensitivity to Ara-C, DNR or Doxo, and enhanced the capacity of colony formation (Figure 2). Inversely, inhibition of c-Myc by shRNA or specific c-Myc inhibitor 10058-F4 rescued the sensitivity to Ara-C, DNR or Doxo treatment in drug resistant cells and reduced the colony formation (Figure 2, 5). In the ATRA-induced differentiation model, c-Myc over-expression reduced CD11b expression, and exhibited a decreased reduction of NBT to formazan (Figure 3), which was associated with C/EBPβ down-regulation (Figure 4). Importantly, in primary AML BMMCs, the expression of *c-Myc* and *C/EBPβ* displayed a strong negative correlation. 10058-F4 showed anti-proliferative activity and increased cellular differentiation with up-regulation of C/EBPβ expression (Figure 6). These results clearly indicated an important role of c-Myc in the drug resistance of AML and demonstrated that c-Myc inhibition could overcome drug resistance in AML treatment.

Several studies reported that high c-Myc expression promoted tumor formation and development *in vitro* and *in vivo* while its down-regulation caused tumor cell growth arrest, enhanced apoptosis, senescence, or differentiation [28]. Importantly, c-Myc expression contributed to leukemogenesis and promoted the leukemia disease progress [13]. c-Myc inhibition prevented leukemia initiation and impaired the growth of relapsed pediatric T-ALL cells [29]. Clinical studies reported that AML patients had significantly higher c-Myc expression than health controls [16]. Our finding showed that the drug-resistant leukemic cells displayed high c-Myc expression with increased colony formation capacity, as shown in Figure 1, which suggested that high level of c-Myc expression might predict poor prognosis. c-Myc over-expression reduced the sensitivity of NB4 cells to Ara-C, DNR or Doxo, and increased cell colony formation ability, while c-Myc inhibition in NB4-R2 cells restored the sensitivity to Ara-C, DNR or Doxo treatment and reduced the colony formation capacity (Figure 2). Thus, c-Myc expression was positively correlated with drug resistance of leukemic cells, and could act as a significant clinical biomarker for AML prognosis.

As a key transcriptional factor, c-Myc decides the cell fate through regulation of a series of downstream genes. c-Myc regulates 15% of all genes expression through binding on Enhancer Box sequences (E-boxes) and recruiting histone acetyltransferases (HATs) [30], [31]. Previous study showed that c-Myc determined transcriptional profiles of ATP-binding cassette (ABC) transporter genes in CML CD34+ hematopoietic progenitor cells, leading to drug efflux and resistance [32]. As well, c-Myc regulated multiple microRNAs, including induction of microRNAs with oncogenic properties and repression of microRNAs with tumor suppressor function. c-Myc increased miR-144/451 expression, contributing to the acquired imatinib resistance in K562 cells [33]. In addition, c-Myc antagonized the differentiation induced by imatinib in CML cells [11]. Of importance, low stage of cell differentiation was associated with poor outcome in tumors [34]. Using the ATRA-induced differentiation model, c-Myc over-expression in NB4 cells maintained poor differentiation, as presenting with decrease of CD11b expression and NBT reduction (Figure 3). Moreover, c-Myc inhibitor 10058-F4 increased CD11b expression in the AML patient BMMCs (Figure 6). These results indicated that c-Myc expression was associated with drug resistance of AML cells through maintaining the state of poor differentiation.

With the characterization of the interplay between c-Myc and downstream targets, the mechanism of c-Myc maintains cell immature state still needs to clarify. Myc down-regulated C/EBP transcription factors was reported previously [35]. It has been shown that c-Myc repressed C/EBPα [36], and interacted with Max and Miz1 to suppress C/EBPδ promoter activity and gene expression [37]. In our study, we found that c-Myc over-expression inhibited the expression of C/EBPβ induced by ATRA treatment. C/EBPβ over-expression could reverse c-Myc induced drug resistance (Figure 4). Importantly, the expression of *c-Myc* was negatively correlated with *C/EBPβ* in primary AML cells (Figure 6). Thus, AML cells might maintain immature or poorly differentiated state to prevent cytotoxicity through C/EBPβ repression by c-Myc. Inhibition of c-Myc in AML could help to reverse the drug resistance of leukemic cells and enhance the treatment efficacy.

The main reason for the poor five-year survival rate in AML patients was that leukemic cells displayed drug resistance to the current drugs, leading to disease relapse. Thus, new approach in AML treatment with specific inhibitor targeting drug resistance related genes was required to reduce AML relapse. Previous reports showed that c-Myc inhibition sensitized the chemotherapeutic agents against different types of tumors, including liver cancer [21], Lewis lung carcinoma [18] and melanoma [38]. In our study, c-Myc inhibitor 10058-F4 promoted AML cells differentiation through increasing the rate of CD11b positive section and restored cell sensitivity to Ara-C, DNR or Doxo (Figure 5, 6), indicating that inhibition of c-Myc activity could suppress the drug resistance to chemotherapeutics in AML cells by promoting cell differentiation. Currently, CX-3453 targeting c-Myc was now in phase II clinical trials (NCT00780663) for neuroendocrine carcinoma. With the wide use of c-Myc inhibitor for different cancers in clinical trials, it should be considerate to apply c-Myc inhibitor in combination therapies to overcome drug resistance, reducing disease relapse.

In conclusion, we found that c-Myc over-expression in AML cells reduced the sensitivity to chemotherapeutic drugs. Inhibition of c-Myc could rescue drug sensitivity by increasing C/EBPβ expression. Taken together, c-Myc inhibition provided a novel strategy to overcome drug resistance in AML treatment.

## Supporting Information

Figure S1 **NB4-R2 and K562/G cells are respectively resistant to ATRA and imatinib.** (A) NB4 and NB4-R2 cells were treated with various concentrations of ATRA for 72 h, and MTT assay was performed. (B) K562 and K562/G were exposed to various concentrations of imatinib for 48 h, and the cell viability was also tested by MTT assay. ***p<0.001, Student's t test.
(TIFF)

Figure S2 **c-Myc over-expression induces drug resistance and high colony formation capacity in U937 cells.** (A) c-Myc was over-expressed in U937 cells (U937/MYC). Cell lysates were subjected to western blotting analysis. (B) The statistical result of colony formation number was shown. (C) U937/GFP and U937/MYC cells were treated with Ara-C (0.1 µM), DNR (0.2 µg/ml) or Doxo (0.04 µg/ml) for 48 h. Drug sensitivity was testified by MTT assay (a–c). (D)U937/GFP and U937/MYC cells were treated with 1 µM ATRA for 72 h. Flow cytometry was performed to determine the expression of CD11b (a), and the percentages of CD11b positive cells were under census (b). (E) Wright-Giemsa staining images of cells were captured by oil immersion lens (magnification, ×1 000). Segmented cells after 72 h of ATRA incubation were annotated by black arrows. (F) NBT reduction assay was performed to clarify the differentiation state. Data summarized three independent experiments. *p<0.05, **p<0.01, ***p<0.001, Student's t test.
(TIFF)
