# Supplementary figures and images for: Inhibition of c-Myc Overcomes Cytotoxic Drug Resistance in Acute Myeloid Leukemia Cells by Promoting Differentiation
Source: PLoS One. 2014 Aug 15;9(8):e105381. doi: 10.1371/journal.pone.0105381 (PMC4134294; doi:10.1371/journal.pone.0105381)

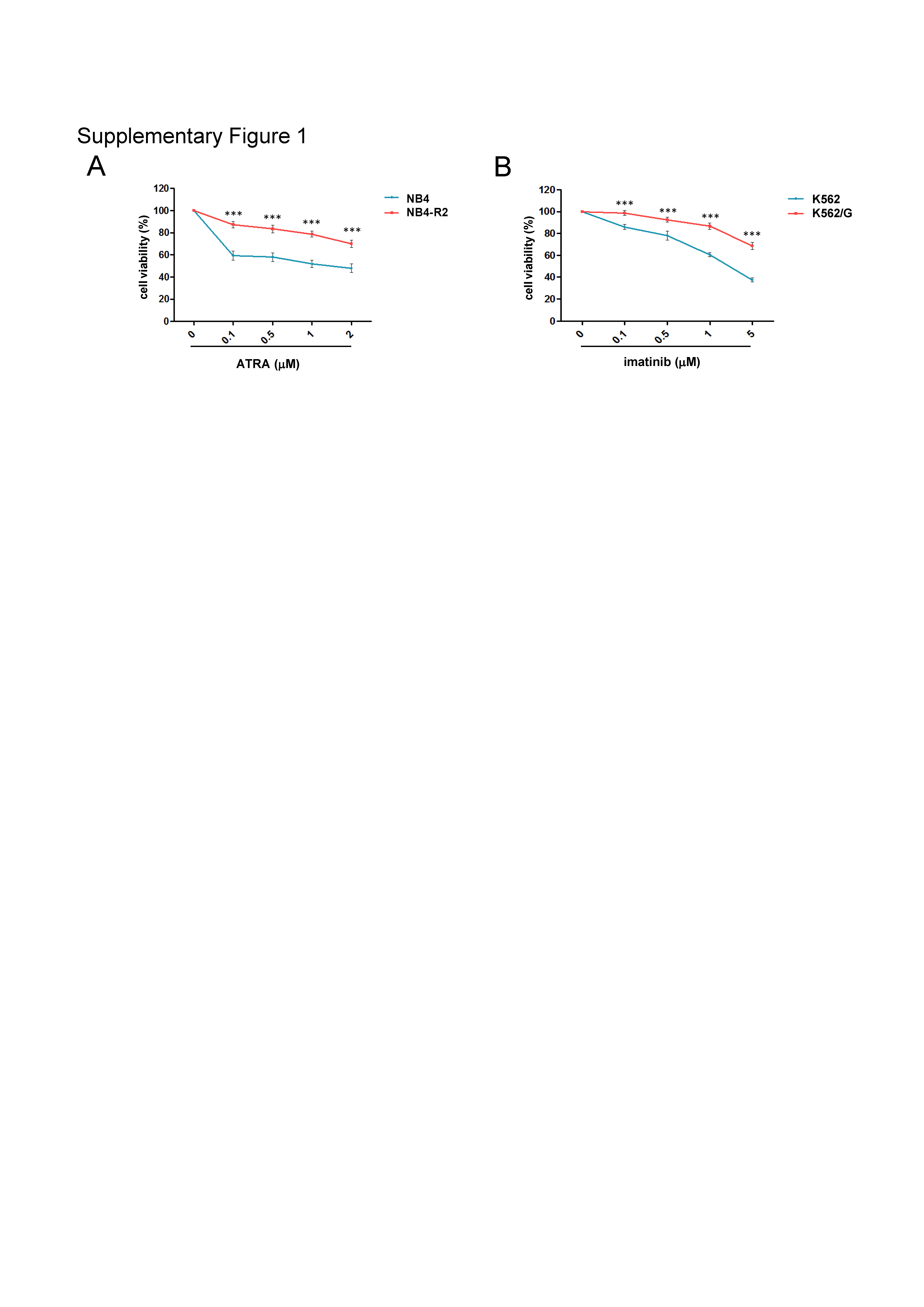

Supplement: Figure S1 — NB4-R2 and K562/G cells are respectively resistant to ATRA and imatinib. (A) NB4 and NB4-R2 cells were treated with various concentrations of ATRA for 72 h, and MTT assay was performed. (B) K562 and K562/G were exposed to various concentrations of imatinib for 48 h, and the cell viability was also tested by MTT assay. ***p<0.001, Student's t test. (TIFF) [file pone.0105381.s001.tiff]

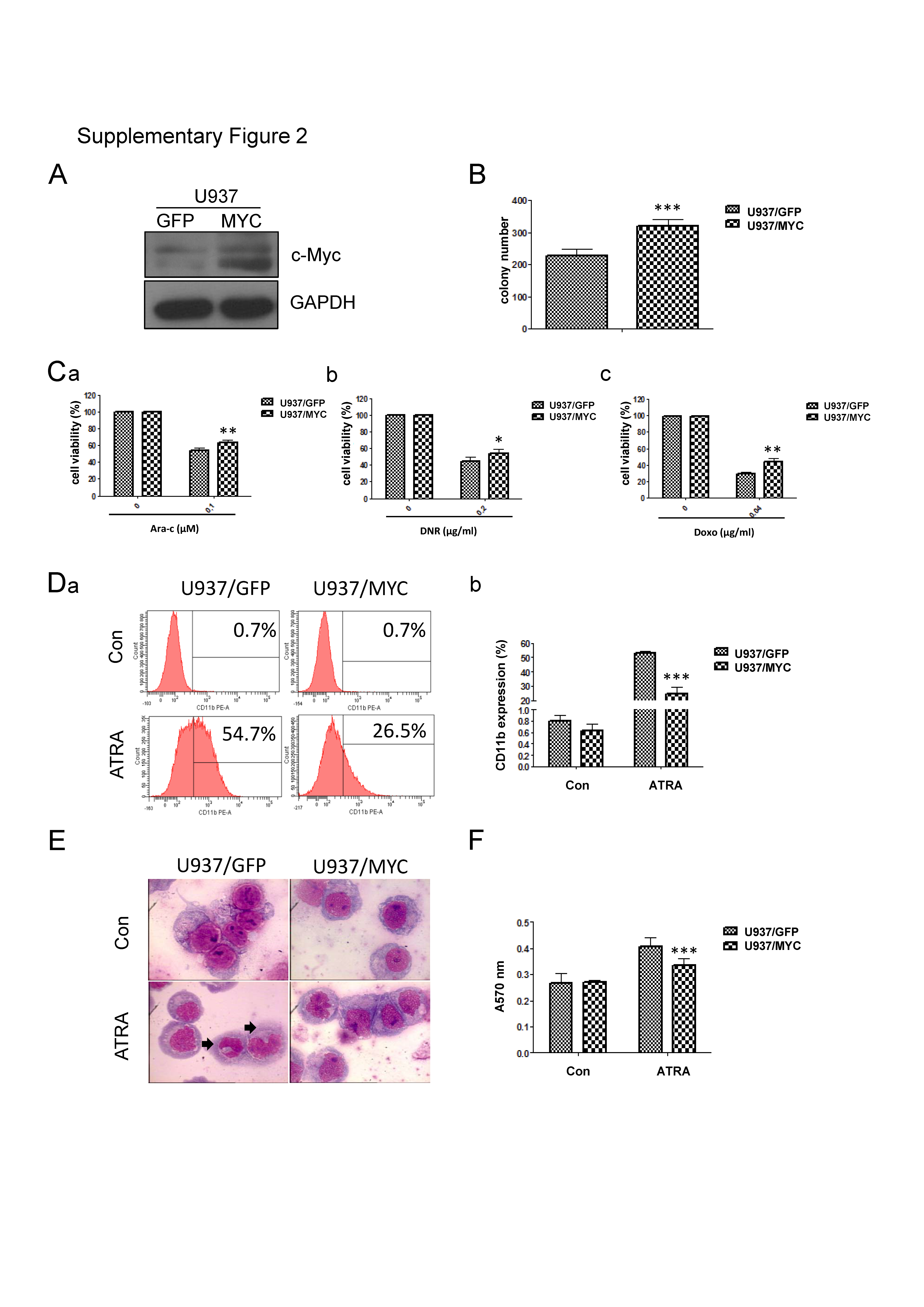

Supplement: Figure S2 — c-Myc over-expression induces drug resistance and high colony formation capacity in U937 cells. (A) c-Myc was over-expressed in U937 cells (U937/MYC). Cell lysates were subjected to western blotting analysis. (B) The statistical result of colony formation number was shown. (C) U937/GFP and U937/MYC cells were treated with Ara-C (0.1 µM), DNR (0.2 µg/ml) or Doxo (0.04 µg/ml) for 48 h. Drug sensitivity was testified by MTT assay (a–c). (D)U937/GFP and U937/MYC cells were treated with 1 µM ATRA for 72 h. Flow cytometry was performed to determine the expression of CD11b (a), and the percentages of CD11b positive cells were under census (b). (E) Wright-Giemsa staining images of cells were captured by oil immersion lens (magnification, ×1 000). Segmented cells after 72 h of ATRA incubation were annotated by black arrows. (F) NBT reduction assay was performed to clarify the differentiation state. Data summarized three independent experiments. *p<0.05, **p<0.01, ***p<0.001, Student's t test. (TIFF) [file pone.0105381.s002.tiff]
